# Chamomile and Urtica dioica extracts improve immunological and histological alterations associated with polycystic ovarian syndrome in DHEA -induced mice

**DOI:** 10.1186/s12906-023-03936-7

**Published:** 2023-04-03

**Authors:** Maryam Shamsi, Ali Ganji, Ghasem Mosayebi, Ensieh Seif Amirhoseiny, Sepideh Shohani, Ali Ghazavi

**Affiliations:** 1 MSc in Histology and Embryology, Arak University of Medical Sciences, Arak, Iran; 2 Department of Immunology, School of Medicine, Arak University of Medical Sciences, Arak, Iran; 3 Molecular and Medicine Research Center, Arak University of Medical Sciences, Arak, Iran; 4 Traditional and Complementary Medicine Research Center (TCMRC), Arak University of Medical Sciences, Arak, Iran; 5 Department of Biotechnology and Molecular Medicine, School of Medicine, Arak University of Medical Sciences, Arak, Iran; 6 Infectious Diseases Research Center (IDRC), Arak University of Medical Sciences, Arak, Iran

**Keywords:** FRAP, Chamomile, Nettle, Polycystic ovary syndrome, Treg

## Abstract

**Background:**

One of the novel mechanisms in the pathogenesis of Polycystic ovary syndrome (PCOS) is low-grade chronic inflammation. Chamomile (Matricaria recutita L.) and Nettle (Urtica dioica), with phytoestrogenic and antioxidant properties, are traditionally used to treat gynecological diseases. This study investigated the immune-modulating effects of these two plants.

**Methods:**

Following the induction of PCOS by subcutaneous injection (SC) of Dehydroepiandrosterone (DHEA) in BALB / C mice. Mice were treated in five groups: Sham, PCOS, PCOS + Chamomile, PCOS + Nettle, and PCOS + Chamomile and Nettle for 21 days. Ovarian morphology, blood antioxidant capacity, the abundance of Treg cells, and expression of matrix metalloproteinase-9 (MMP-9), transforming growth factor-ß (TGF-ß), cyclooxygenase-2 genes (COX-2), and tumor necrosis factor-alpha (TNF-α) were measured.

**Results:**

Folliculogenesis, Cystic follicles, and corpus luteum improved in the treatment groups (P < 0. 05). Treg cells in the DHEA group were significantly reduced compared to the Sham group (P < 0. 01). However, this decrease was not corrected in treatment groups (P > 0. 05). Total serum antioxidant capacity was significantly increased in the treatment group of Nettle and Chamomile + Nettle (P < 0. 05). The expression of MMP9 and TGFβ genes in the PCOS group was significantly higher than the Sham group (P < 0. 05), which the expression of MMP9 was corrected by treatment with Chamomile + Nettle extract (P < 0. 05).

**Conclusion:**

Chamomile and Nettle extract may be an effective supplement in improving the histological and immunological changes of PCOS. However, more research is needed to confirm its effectiveness in humans.

## Background

One of the main causes of infertility in women is Polycystic ovary syndrome (PCOS), with a prevalence of 6–21% of reproductive age. The disease is characterized by hyperandrogenism, persistent anovulation, Polycystic ovary morphology (PCOM), and insulin resistance and affects some body systems, including the endocrine, metabolic, and reproductive systems [1–3].

PCOS is a multifactorial disorder; Different factors have been identified in the pathogenesis of this disease. The interaction between environmental factors, growth factors, and various genetic factors is involved in causing this disease. Another factor involved in the pathogenesis of this disease is immune system disorders, which are associated with an increase in several inflammatory factors and mediators, including leukocytes, cytokines, and reactive oxygen species (ROS) [4, 5]. Balance in the amount of inflammatory factors is necessary for ovarian function. Their imbalance causes ovarian dysfunction such as ovulation, folliculogenesis, hormone balance and corpus luteum function. In patients with PCOS, the increase of inflammatory markers such as TNF-α, IL-1β, IL-17, MCP-1, and Macrophage-1α (MIP-1α) causes oxidative stress and dysfunction of endothelial cells [6–8]. These inflammatory reactions are associated with obesity, hyperandrogenism, insulin resistance, and type 2 diabetes, ultimately increasing infertility by affecting physiological processes such as ovulation [9–11]. The use of drugs and assisted reproductive techniques (ART) is a common treatment for PCOS. Studies show that due to the high price of chemical drugs, drug resistance, and widespread side effects, Medicinal plants with antioxidant and anti-inflammatory properties can be an excellent alternative to chemical drugs and can be used as an effective drug in the management and treatment of PCOS. Therefore, the World Health Organization (WHO), encouraging researchers to use herbal medicines rationally, recommends their use as a new source of treatment. Chamomile and Nettle, with phytoestrogenic and antioxidant properties, are traditionally used to treat gynecological diseases [12–15].

Chamomile (Matricaria recutita L.), from the Asteraceae family, is one of the well-known herbs. This plant has various medicinal properties, including anti-inflammatory, anti-diabetic, anti-hepatotoxic, anti-cancer, anti-viral, and antioxidant. These benefits are partly due to the most important ingredients in Chamomile, including phenolic, flavonoids, and phytoestrogens [16]. It has been reported that Chamomile has phytoestrogenic and antioxidant properties that can improve ovarian histological changes and reduce luteinizing hormone (LH) and Follicle-stimulating hormone(FSH), estradiol, testosterone levels in PCOS, and improve PCOS symptoms [17–19].

Nettle (Urtica Dioica L.) is extensively used as traditional medicine, belonging to the Urticaceae family. Nettle has gained attention in the scientific community for its anti-diabetic, anti-inflammatory, Anti-viral, anti-ulcer, immunological stimulatory, anti-infectious, and antioxidant properties [20, 21]. The Nettle’s biological activities are assigned to this plant’s major components, including flavonoids, phenolic acid, lignans, and phytosterols [22, 23]. Studies have shown that Nettle extract reduces morphological and histological alterations in PCOS [24, 25]. Due to the antioxidant, phytoestrogens, and anti-inflammatory properties of Nettle and Chamomile, in this study, we expected combining Chamomile and Nettle to reduce the adverse effects of PCOS in mice. Therefore, this study investigated the combined effects of Chamomile and Nettle on PCOS mice.

## Materials and methods

Experimental protocols were conducted according to the Iranian animal ethics framework and were approved by the Institutional Ethics Committee of the Arak University of Medical Sciences (IR.ARAKMU.REC.1398.243).

### Extraction

Fresh Chamomile and Nettle plants have been obtained from a local herbal market in Arak (Iran). The herbarium has been scientifically approved by the School of Medicine, Arak University of Medical Sciences. All Chamomile and Nettle leaves were washed and dried, and after pulverizing the dried leaves, methanolic extract of Chamomile and Nettle was obtained by the Maceration method. 100 g of Chamomile powder in 50% methanol and 100 g of Nettle powder in 20% methanol were soaked for 24 h to prepare the extract. The extract was filtered by Whatman filter paper 0.45 μm and 0.22 μm, and the solvent was vacuum-distilled at 60 °C in a rotary evaporator (at 60ºC) (Heidolph, Germany). After removing the solvent, the extract was dried at 40ºC and stored at 4ºC following weighing and calculating the yield [26, 27]. All experimental protocols were conducted according to the ethical guidelines/regulations governing the use of plants.

### Acute toxicity assessment

Before starting the study, ED50 (mean effective dose) of Chamomile and Nettle extracts was evaluated. According to the OECD (Organization for Economic Co-operation and Development) guidelines for measuring acute toxicity, five BALB/C female mice (5 to 6 weeks old) were randomly assigned to each group to assess acute toxicity. The sham group received only solvent (sesame oil) intraperitoneal injection (IP). The Chamomile group received 500 mg/kg of chamomile extract for 14 days. The Nettle group received 250 mg/kg of Nettle extract for 14 days, and the Chamomile + Nettle group received 500 mg/kg of Chamomile extract + 250 mg/kg of Nettle extract for 14 days. The injection volume was 10 ml/kg. Initially, mice in the first 6 h after injection and then daily for 14 days in terms of mortality, weight changes, behavioral pattern, physical appearance (skin and fur), changes in respiration, injury, pain, and symptoms (tremor, Convulsions, Diarrhea, Lethargy) were monitored. The weight of mice was also calculated on the first, seventh, and fourteenth days [28].

### Experimental animals

In this study, 30 immature BALB / C mice (21 days old and weighing about 14–15 g) were purchased from the Pasteur Institute of Iran (IPI, Tehran). The animals were grouped in a well-ventilated room at a temperature (23 ± 1 ° C) and (12-hour light / 12-hour dark) cycle. All mice were fed the standard laboratory feeding platform, and Ad Libitum fresh water was available. Mice with at least two consecutive 4-day estrus cycles were eligible for the study. Selected mice were randomly divided into five groups: (n = 6 / group)

(1) Sham group (0.1 ml of sesame oil (Barij Esans Iran) was given subcutaneously for 21 days), (2) PCOS group (mice with DHEA-induced PCOS), (3) Chamomile treatment group (received Chamomile extract at a concentration of 500 mg/kg for 21 days by IP injection for the treatment of PCOS), (4) Nettle treatment group (received Nettle extract at a concentration of 250 mg/kg for 21 days by IP injection for the treatment of PCOS), (5) Chamomile + Nettle treatment group, (received Chamomile extract at a concentration of 500 mg/kg + Nettle extract at a concentration of 250 mg/kg for 21 days by IP injection for the treatment of PCOS).

PCOS mice received 60 mg/kg DHEA (Sigma America) in 0.1 ml of sesame oil subcutaneously for 21 days. In the treatment groups, the animals were weighed every 3 to 4 days using the scale (Mettler Toledo Suisse).

### Evaluation of the estrous cycle

In mice, the sexual cycle lasts 4 to 5 days. The estrous cycle stages were determined during the treatment period by vaginal smear. Vaginal smears were prepared daily between 9 am and 10 am. three 7-day periods (the first period was one week after receiving DHEA, the second period was the last week of receiving DHEA, and the third period was the last week of treatment with Chamomile+/Nettle extract) from mice and stained with trypan blue. Four phases, proestrus, estrus, diestrus, and metestrus, were observed under a microscope [29].

### Sample collection

At the end of treatment (24 h after the last injection), mice were anesthetized in the morning fasting by IP of Ketamine-xylazine(Fish et al. 2008). Blood samples were taken immediately from the heart. Serum obtained after separation was stored at -70 ° C to measure Serum antioxidant capacity (FRAP) and fasting blood glucose (FBG) tests. The left ovaries were fixed at 10% formaldehyde to evaluate ovarian histomorphology (Merck, Germany). Spleens were dissected to count Treg cells. In addition, adipose tissue around the right ovary and uterus was removed to examine the expression of genes involved in inflammation.

### Ovarian histomorphology

For histological Analysis, the left ovary was detached from the abdominal cavity of the mice and rinsed with saline solution, then placed in 10% formaldehyde. Paraffin-embedded ovaries were serially incised to a thickness of 7 μm. Then, the sections were paraffinized with xylene, rehydrated with a descending series of ethanol, and finally stained with hematoxylin and eosin (H&E) and viewed under a light microscope with a magnification of 40×. The follicles were counted according to the methods described by Lou et al., and the number of Preantral oocytes, Antral, Cystic follicles and Corpus luteum (CL) was counted [30].

### Fasting blood glucose (FBG) measurement

Mice fasted for 12 h with access to water. Glucose concentration was measured by enzymatic method using glucose oxidase colorimetric kit (Zist Shimi, Tehran, Iran).

### FRAP activity assay

Serum antioxidant capacity measured by the FRAP method. This method is prepared by mixing 300 mM acetate buffer, 10 mM TPTZ solution (Sigma–Aldrich, St. Louis, MO, USA), in 40 mM HC1, and ferric chloride solution. FRAP reagents were mixed with samples and incubated for 10 min at 37 ° C. Then, the light absorption of the samples at 593 nm was read by ELISA Reader, and the concentration of the samples was determined using a standard curve.

### Treg cells frequency

In this experiment, Splenocytes were stained with PerCP-labeled anti-mouse CD25, PE-labeled anti-mouse CD4, and FITC-labeled anti-mouse FoxP3. Data using a BD FACSCalibur (BD Biosciences, CA, USA), and then Analysis was performed by FlowJo software (Tree Star, Inc., OR, USA) with 20,000 cells.

### Gene expression assay

The adipose tissue surrounding the right ovary and uterus was separated and homogenized in the RNA Lysis buffer. Then, after extracting total RNA and synthesizing cDNA(Yekta Tajhiz, Iran), the primers of the target genes and a housekeeping gene (GAPDH) were designed using Allele ID 6 software (Premier Bio soft, USA) and aligned on BLAST website (Table 1). Real-time PCRs were run using SYBR Green Master mix according to the protocols (Yekta Tajhiz, Iran) on a LightCycler® 96 thermal cycler Instrument (Roche Diagnostics, Switzerland). Amplification specificity was checked using a melting curve assay.

**Table 1:** PCR Primer Sequences

Target	Amplicon (bp)	Primers	Sequences, 5’→ 3’
GAPDH	224	F	CGGTGTGAACGGATTTGG
		R	CTCGCTCCTGGAAGATGG
TNF-α	201	F	CCTCTTCTCATTCCTGCTTGTG
		R	ACTTGGTGGTTTGCTACGAC
TGF-β	193	F	AATTCCTGGCGTTACCTTGG
		R	GGCTGATCCCGTTGATTTCC
COX-2	248	F	GCACTACATCCTGACCCACTTC
		R	GCTCCTTATTTCCCTTCACACC
MMP-9	249	F	GGCGTGTCTGGAGATTCG
		R	GCAGGAGGTCGTAGGTCAC

GAPDH: glyceraldehyde-3-phosphate dehydrogenase; TNF-α: Tumor necrosis factor-alpha; TGF-β: transforming growth factor-β; COX-2: Cyclooxyganase-2; MMP-9: Matrix metallopeptidase-9; F: Forward; R: Reverse.

### Statistical analysis

All statistical analyses were done using SPSS software. The one-way Analysis of variance (ANOVA) and Scheffe’s post hoc test was used to compare the determination of statistical significance between the experimental groups. Results were reported as (Mean ± SD). p < .05 was considered significant.

## Results

### Acute toxicity results

By administering a concentration of 500 mg/kg of Chamomile extract and + 250 mg/kg of Nettle extract, no change in the physical appearance of mice (skin and fur) and respiration was observed. The mice had no symptoms (tremor, seizures, diarrhea, and lethargy). The mean weight increased in all treatment groups in the first, seventh, and fourteenth days, but this increase was not significant. Therefore, the concentration of 500 mg/kg of Chamomile extract as the therapeutic concentration of the Chamomile group and the concentration of 250 mg/kg of Nettle extract as the therapeutic concentration of the Nettle group. The concentration of 500 mg/kg of Chamomile extract + concentration of 250 mg/kg of Nettle extract as therapeutic concentration Chamomile + Nettle group was selected.

### Stress cycle

**Fig. 1:**
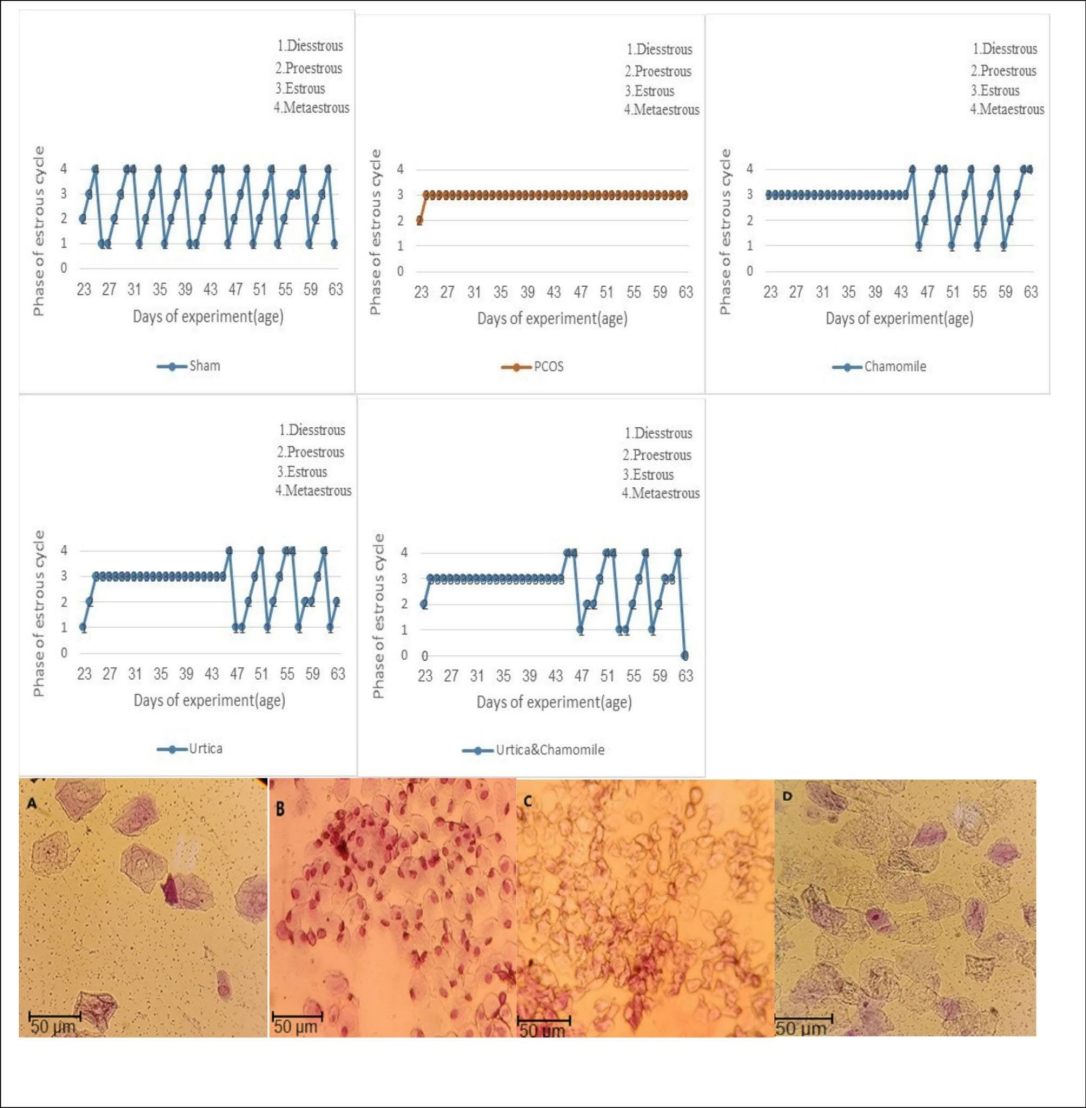
The effect of Chamomile, Urtica, Chamomile& Urtica on estrous cycle in experimental groups. Data show the most represented phase of the estrous cycle in each group. Sham group: mice were receiving 0.1 ml of sesame oil; PCOS group: mice with DHEA-induced PCOS; Chamomile group: receiving Chamomile extract at a concentration of 500 mg/kg; Urtica group: receiving Urtica extract at a concentration of 250 mg/kg; Chamomile + Urtica group: receiving Chamomile extract at a concentration of 500 mg/kg + Urtica extract at a concentration of 250 mg/kg. Vaginal smears of experimental groups showing diestrous stage (**A**), proestrus (**B**), estrous stage (**C**), and metestrus (**D**) were observed (trypan blue stain, X40). *P* = Proestrus, *E* = Estrous, *M* = Metestrus, and *D* = Diestrous.

The estrous cycle of the control and PCOS groups is presented in (Fig. 1). The estrous cycle was regular in the control group. After the induction of PCOS, the estrous cycle was disrupted, and the mice were in the estrous phase. The presence of epithelial horn cells indicates the formation of cystic follicles in the ovary. Nettle and Chamomile treatment groups restored the estrous cycle to normal (Fig. 1).

### Ovarian morphology

**Fig. 2:**
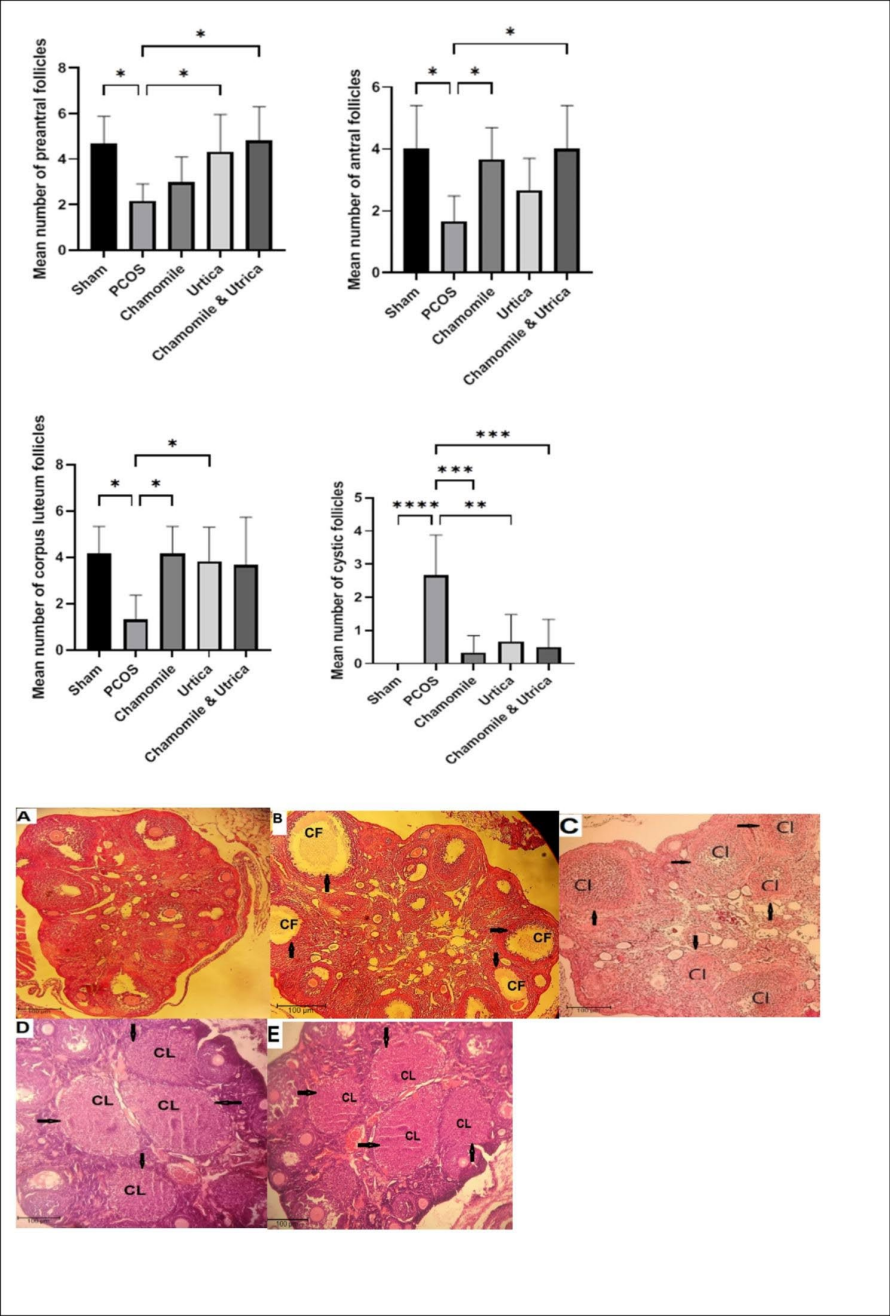
effect of Chamomile, Urtica, Chamomile& Urtica on the number of different follicles in experimental groups. Sham group: mice were receiving 0.1 ml of sesame oil; PCOS group: mice with DHEA-induced PCOS; Chamomile group: receiving Chamomile extract at a concentration of 500 mg/kg; Urtica group: receiving Urtica extract at a concentration of 250 mg/kg; Chamomile + Urtica group: receiving Chamomile extract at a concentration of 500 mg/kg + Urtica extract at a concentration of 250 mg/kg. The representative histological photomicrographs of ovaries of mice. **A** Sham; **B** PCOS; **C** Urtica; **D** Chamomile; **E** Chamomile& Urtica. The sham group showed normal ovarian morphology, whereas the PCOS group exhibited many cystic and atretic follicles. Treatment groups indicated increasing the number of corpus luteum, preantral, and antral follicles and decreasing the number of cystic follicles. CL, corpus luteum, CF, cystic follicles. (40X magnification, H&E). Dates are expressed as mean ± SEM.( n = 6). * P < .05, ***: P-value < 0.001

Data on the number of different follicles are presented in (Fig. 2). in the sham group, the ovary shows normal follicles with preantral, antral, and corpus luteum follicles. After induction of PCOS with DHEA, the number of preantral (P = .01), antral (P = .01), and corpus luteum follicles (P = 0. 01) in mice decreased significantly, and the number of cystic follicles in this group increased significantly (P < 0. 0001).

Treatment with Nettle extract significantly increased the number of preantral follicles (P = .04) and corpus luteum (P = 0. 04) compared to the PCOS group and decreased the number of cystic follicles (P = 0. 001). However, antral follicles were not significantly different from the PCOS group (P = 0. 58).

In the Chamomile group, the number of cystic follicles decreased compared to the PCOS group (P = 0. 0002), and also the antral (P = .04) and corpus luteum follicles (P = 0. 01) increased. However, the number of preantral follicles was not significantly different from the PCOS group (P = 0. 78).

Combination treatment with Chamomile and Nettle extracts increased periantral (P = .01) and antral follicles (P = .01) and decreased cystic follicles (P = 0. 0006) compared to the PCOS group. However, the corpus luteum was not significantly different from the PCOS group (P = 0. 06). There was no significant difference between ovarian follicles in the treatment subgroups (P > 0.05) (Fig. 2).

### Serum glucose levels

**Fig. 3:**
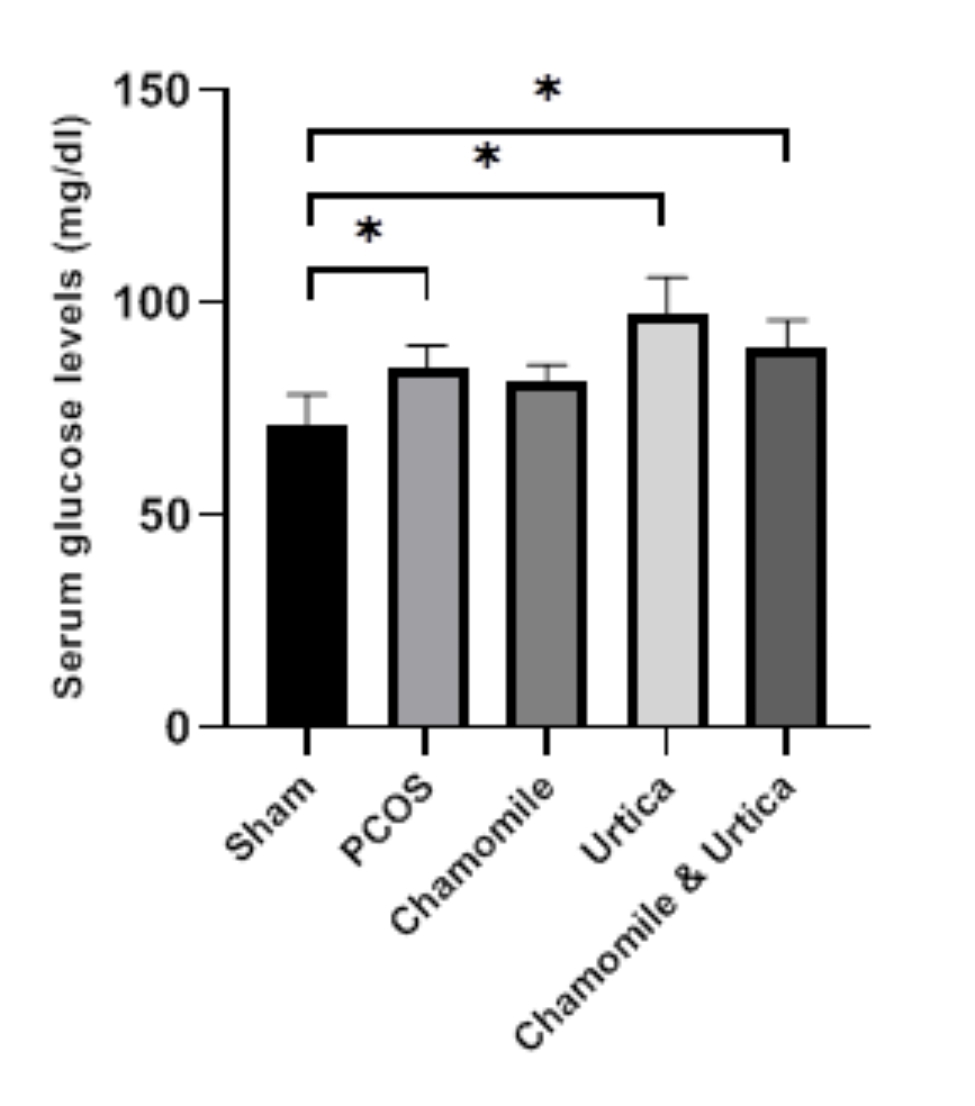
The effect of Chamomile, Urtica, Chamomile& Urtica on serum glucose levels in experimental groups. Sham group: mice were receiving 0.1 ml of sesame oil; PCOS group: mice with DHEA-induced PCOS; Chamomile group: receiving Chamomile extract at a concentration of 500 mg/kg; Urtica group: receiving Urtica extract at a concentration of 250 mg/kg; Chamomile + Urtica group: receiving Chamomile extract at a concentration of 500 mg/kg + Urtica extract at a concentration of 250 mg/kg. Values are expressed as mean ± SEM.( n = 6). * P < .05

Data on serum glucose levels are shown in (Fig. 3). Our results showed that induction of PCOS in mice significantly increased blood glucose levels. (P = 0. 01)

In the treatment groups of Nettle, a significant increase was observed compared to the Sham group (P < 0. 0001) and PCOS group (P = 0. 02). Also, in the Chamomile + Nettle group, a significant increase was observed compared to the Sham group (P = 0. 0007). Other treatment groups were not significantly different from the PCOS group (P > 0. 05).

### FRAP assay

**Fig. 4:**
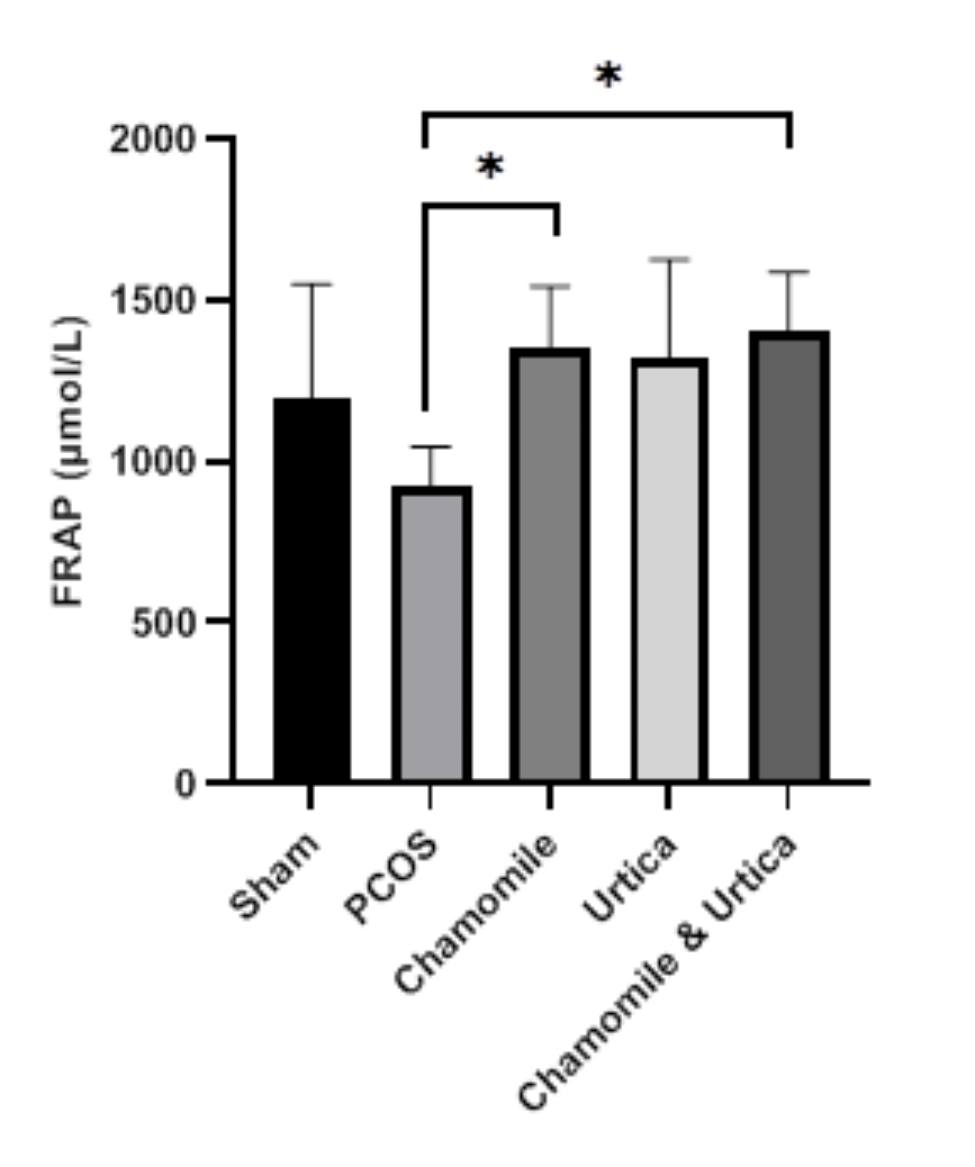
The effect of Chamomile, Urtica, Chamomile& Urtica on FRAP in experimental groups. Sham group: mice were receiving 0.1 ml of sesame oil; PCOS group: mice with DHEA-induced PCOS; Chamomile group: receiving Chamomile extract at a concentration of 500 mg/kg; Urtica group: receiving Urtica extract at a concentration of 250 mg/kg; Chamomile + Urtica group: receiving Chamomile extract at a concentration of 500 mg/kg + Urtica extract at a concentration of 250 mg/kg. Values are expressed as mean ± SEM.( n = 6). * P < .05

Figure 4 shows the therapeutic effects of Nettle and Chamomile on FRAP antioxidant status. According to the results, the PCOS group had the lowest serum FRAP. However, this decrease was not significant (P = 0. 3). The use of Chamomile (P = 0. 04) and Chamomile + Nettle (P = 0. 01) extracts increased serum FRAP levels against the PCOS group. However, Nettle did not affect PCOS (P = 0. 07). There was also no significant difference between the treatment groups (P > 0. 05).

### Number of Treg cells

**Fig. 5:**
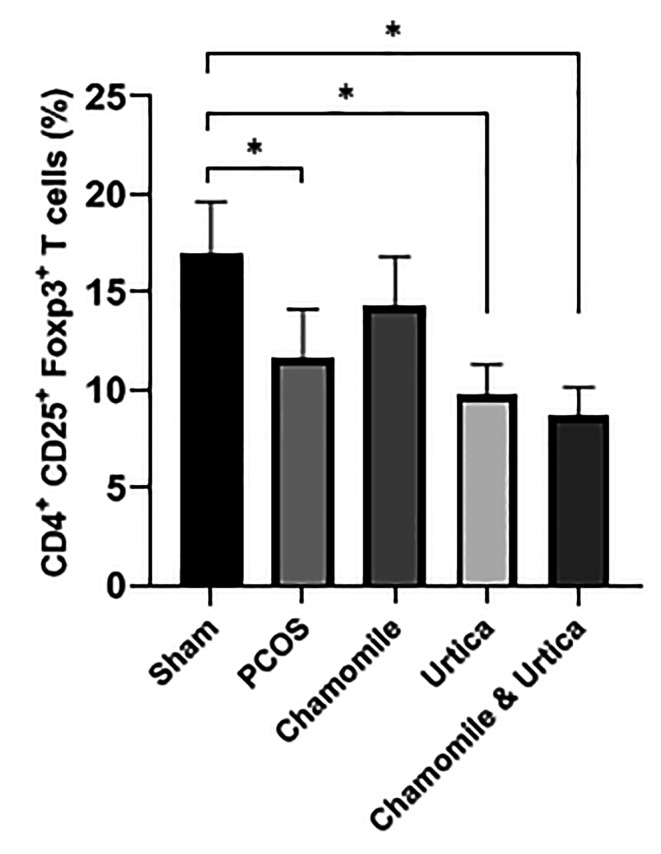
The effect of Chamomile, Urtica, Chamomile& Urtica on the number of Treg cells in experimental groups. Sham group: mice were receiving 0.1 ml of sesame oil; PCOS group: mice with DHEA-induced PCOS; Chamomile group: receiving Chamomile extract at a concentration of 500 mg/kg; Urtica group: receiving Urtica extract at a concentration of 250 mg/kg; Chamomile + Urtica group: receiving Chamomile extract at a concentration of 500 mg/kg + Urtica extract at a concentration of 250 mg/kg. Values are expressed as mean ± SEM.( n = 6). * P < .05

The data related to Treg cells are presented in (Fig. 5). The results show that the number of Treg cells after induction of PCOS was significantly reduced compared to the Sham group (P = 0. 007). Treatment with Nettle extract and Chamomile + Nettle also decreased significantly compared to the Sham group (P < 0. 001), but the treatment groups were not significantly different from the PCOS group (P > 0. 05).

### Evaluation of gene expression of inflammatory markers

**Fig. 6:**
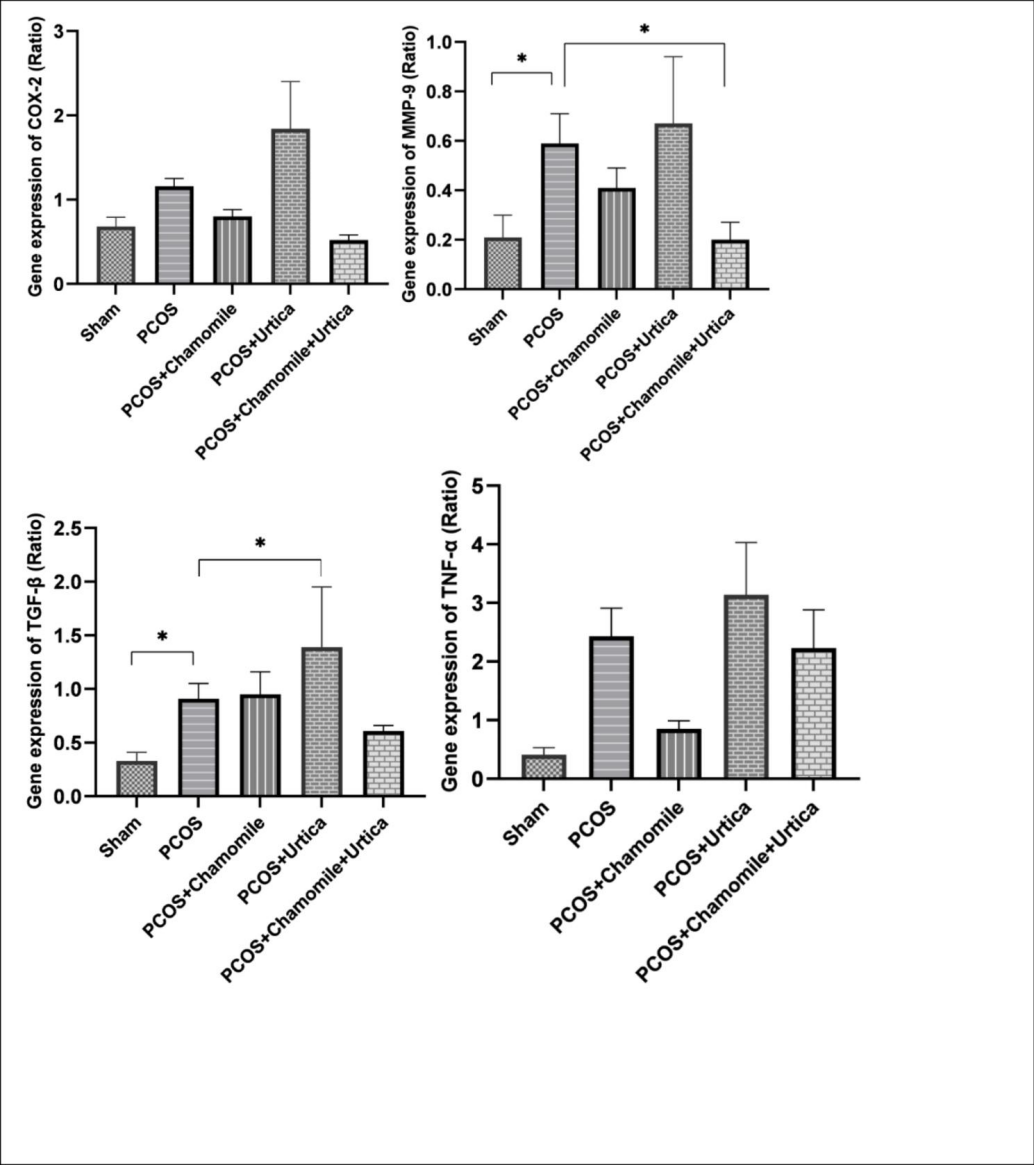
The effect of Chamomile, Urtica, Chamomile& Urtica on gene expression of inflammatory markers in experimental groups. Sham group: mice were receiving 0.1 ml of sesame oil; PCOS group: mice with DHEA-induced PCOS; Chamomile group: receiving Chamomile extract at a concentration of 500 mg/kg; Urtica group: receiving Urtica extract at a concentration of 250 mg/kg; Chamomile + Urtica group: receiving Chamomile extract at a concentration of 500 mg/kg + Urtica extract at a concentration of 250 mg/kg. Values are expressed as mean ± SEM.( n = 6). * P < .05

The results of expression of inflammatory markers (Fig. 6) showed that induction of PCOS caused a significant increase in inflammatory factors TGF-ß (P = 0. 001) and MMP-9 (P = 0. 042) in the PCOS group compared to the Sham group. Induction of PCOS also increased the expression of COX-2 (P = 0. 22) and TNF-α (P = 0. 09) inflammatory marker genes, But this increase was not significant. Combination treatment with Chamomile + Nettle extract significantly reduced MMP-9 gene expression compared to the PCOS group (P = 0. 049). Treatment with Nettle extract also significantly increased MMP-9 gene expression compared to the PCOS group (P = 0. 01). There was also no significant difference between the treatment groups (P > 0. 05).

## Discussion

In the present study, the combined effects of Chamomile and Nettle on PCOS mice were investigated for the first time. DHEA was used to induce PCOS in this study. After 21 days of DHEA injection, mice showed irregular estrous cycles, characterized by cornified cells, representing cystic follicles and ovarian structures similar to PCOS [31]. Irregular estrous cycles, the presence of cystic follicles in the PCOS group, and a decrease in corpus luteum compared to the Sham group indicate DHEA-induced PCOS in them. Similar results showed an increase in cystic follicles and a decrease in ovarian follicles after DHEA induction in female mice [32, 33].

In our results, induction of PCOS resulted in decreased preantral, antral, and corpus luteum follicles, while cystic follicles increased significantly. The PI3K-Akt Signaling Pathway plays a role in the growth of ovarian follicles, so disruption of the PI3K-Akt Signaling Pathway in PCOS is associated with disruption of follicle growth, hormone synthesis and inflammation [34, 35]. Restuccia et al.‘s study showed that ovarian cysts were formed by eliminating Akt2[36]. In some studies, drugs that inhibit the signalling pathway PI3K-Ak are effective in treating PCOS [37].

Studies have shown that oxidative stress, by producing reactive oxygen species (ROS), can cause DNA damage and lead to excessive proliferation of ovarian cells, ovarian cysts, and infertility [38]. ]. ROS controls physiological activities, including folliculogenesis, steroidogenesis, and corpus luteum function. Therefore, inadequate antioxidant defence and increased ROS can affect reproductive function, and it can involve in the pathogenesis of PCOS by increasing cystic follicles and follicular atresia [39]. Also, changes in ovarian folliculogenesis and cystic follicle formation in PCOS can be associated with hyperandrogenism in this syndrome [40]. Increased ROS and hyperandrogenism in this syndrome cause an increase in ovarian cystic follicles and follicular atresia. In this study, the treatment of mice with Nettle and/or Chamomile improved follicular atresia, which represents the effect of these plants on the maintenance of ovarian folliculogenesis. This may be due to the anti-androgenic activity of Nettle and the phytoestrogenic properties of Chamomile, which improved ovarian folliculogenesis in mice with PCOS.

Previous studies have shown that Nettle extract, with its direct role in increasing estrogen, increases ovarian follicles and can positively affect folliculogenesis, which indicates an improvement in ovarian function. t increase of nitric acid causes dilation of blood vessels and an increase in blood flow to the tissues. Nettle can reduce follicular atresia by increasing the blood flow to the ovaries and then increasing the penetration of oxygen to the granulosa cells [24, 41, 42].

In addition, similar studies have shown that Chamomile, with its phytoestrogens and antioxidant properties, reduces FSH levels of testosterone, oxidative stress, estradiol, and LH and improves ovarian histological changes in mice with PCOS by disappearing cysts and increasing the number of follicles [17, 43]. As a result, based on the study’s findings, it seems that the single and combined use of these two extracts can improve ovarian function changes.

Studies have shown that imbalances between ROS and antioxidant defence systems, including glutathione peroxidase, superoxide dismutase, and catalase enzymes, lead to oxidative stress, which increases oxidative stress (OS) and decreases Significant serum antioxidants are associated with the pathological properties of PCOS [44], so the use of herbs and supplements with antioxidant properties helps reduce oxidative stress [45].

According to FRAP statistical analysis, the total antioxidant capacity in the PCOS group was lower than in the Sham group; however, this decrease was not significant. Chamomile and chamomile + Nettle extract treatment groups increased total antioxidant capacity in PCOS mice.

Chamomile can improve the antioxidant defence system due to the antioxidant agents of apigenin, luteolin, and quercetin. Also, it is involved in increasing antioxidant enzymes such as superoxide dismutase and catalase, which catalyzes the conversion of superoxide radicals to ordinary molecules such as oxygen and hydrogen peroxide [46]. Therefore, this increase in total antioxidant capacity in Chamomile and Chamomile + Nettle groups can be attributed to the antioxidant properties of Chamomile, which was consistent with the study of Al-Ahmadi et al., Who showed, Chamomile increases antioxidant capacity and reduces OS in mice with PCOS [19].

Blood glucose levels were significantly higher in PCOS-induced mice; surprisingly, Chamomile and/or Nettle consumption increased blood glucose in mice. The cause of this effect needs to be better understood and further study. One reason for the increase in blood glucose levels in therapeutic groups affected by Nettle can be the sex and estrogen of animals. In parallel with our findings, non-ovariectomized rats treated with Nettle had higher blood glucose than the ovariectomized group, so Nettle had a diabetic effect on them [39]. It seems Nettle extract blocks estrogen receptors. Also, an increase in blood glucose levels may be associated with insulin resistance syndrome in them [47–49].

Angiotensin II and aldosterone at the cell surface increase oxidative stress and reduce glucose transport by altering insulin controls, thereby increasing insulin resistance. In human and animal models, Renin-angiotensin-aldosterone (RAAS) system inhibitors improve glucose homeostasis by affecting glucose metabolism and insulin resistance and improving the insulin response [50].

As a result, Nettle extract may increase angiotensin II activity in target organs while decreasing insulin sensitivity; therefore, it raised blood sugar levels in non-ovarian female rats, indicating that this hypothesis requires further investigation.

In this research, Treg cells in the PCOS group significantly decreased compared to the sham group. Treg cells CD4 (+) CD25 (+) FOXP3 (+) are a population of Treg cells that are very important for reproductive function. These cells are affected by the androgen, estrogen, and progesterone through their surface receptors, And during the embryonic period and ovulation, their number increases [51–53]. Therefore, the hormonal changes in PCOS play an important role in the dynamic change of Treg cells. Similar studies have been performed on people with PCOS, Showing a significant decrease in Treg cells in these patients compared to healthy individuals [54, 55]. It can be concluded from the results of this study that the reduction of vascular cells is involved in the pathogenesis of PCOS and the Chamomile + Nettle treatment group significantly reduced these cells compared to the sham group.

Numerous studies show that PCOS is related to chronic inflammation and oxidative stress [38, 56]. Ovarian macrophages are one of the influential factors in ovarian inflammation that, by producing cytokines and chemokines, can affect the processes of folliculogenesis, ovulation, and corpus luteum formation [57]. Therefore, several studies have investigated the level of cytokines in PCOS, so the results of previous studies have shown an increase in the inflammatory markers COX2, MMP-9, TNF, and TGF-β in PCOS patients. Our findings were consistent with previous findings on MMP-9 and TGF-β[58–61].

MMPs are a metalloprotease matrix produced by the ovaries; their high level affects ovulation and fertility and causes abnormal follicular atresia and increased ovarian stromal tissue [60].

TGF-β is a cytokine expressed in the ovary and is involved in the pathogenesis of abnormal follicle growth and hyperandrogenism in PCOS [62, 63].

The authors could not find a study that showed a combined effect of Chamomile and Nettle on inflammatory factors MMP-9. However, based on the information obtained from the properties of Nettle and Chamomile in this study, it may be concluded that the combination of Nettle and Chamomile showed better anti-inflammatory and antioxidant properties. It significantly inhibits the expression of MMP. Due to its antioxidant and anti-inflammatory effects, it can regulate inflammatory mediators in PCOS model mice induced by androgen-dehydroepiandrosterone due to its high antioxidant and anti-inflammatory properties [16, 23].

Co-administration of Nettle and Chamomile and their combination may be a proper treatment for reducing the negative effects of PCOS. It is suggested that different doses be used in future research.

## Conclusion

The finding of this study showed that PCOS had a negative impact on ovarian folliculogenesis, blood glucose, Treg cells, and expression of MMP-9 and TGF-β inflammatory marker genes. Nettle and Chamomile extract may be an effective supplement in the histological and immunological improvement of PCOS, although more research is needed to confirm its effectiveness in humans.

## Data Availability

The datasets generated during and/or analyzed during the current study are available from the corresponding author upon reasonable request.

## Abbreviations

PCOS: Polycystic ovary syndrome
SC: subcutaneous injection
DHEA: Dehydroepiandrosterone
MMP-9: matrix metalloproteinase-9
TGF-ß: transforming growth factor-ß
COX-2: cyclooxygenase-2 genes
TNF-α: tumor necrosis factor-alpha
PCOM: Polycystic ovary morphology
ROS: reactive oxygen species
ART: assisted reproductive techniques
WHO: World Health Organization
LH: luteinizing hormone
FSH: Follicle-stimulating hormone
OECD: Organization for Economic Co-operation and Development
IP: intraperitoneal injection
FRAP: Serum antioxidant capacity
FBG: fasting blood glucose
H&E: hematoxylin and eosin
CL: Corpus luteum
GAPDH: glyceraldehyde-3-phosphate dehydrogenase
F: Forward
R: Reverse
P: Proestrus
M: Metestrus
D: Diestrous
ROS: reactive oxygen species
OS: oxidative stress
RAAS: Renin-angiotensin-aldosterone

## References

[1] Rotterdam ESHRE/ASRM-Sponsored PCOS consensus workshop group. Revised 2003 consensus on diagnostic criteria and long-term health risks related to polycystic ovary syndrome (PCOS). Hum Reprod. 2004;19(1):41–7. 10.1093/humrep/deh098 14688154

[2] Armanini D, Boscaro M, Bordin L, Sabbadin C. Controversies in the pathogenesis, diagnosis and treatment of PCOS: focus on insulin resistance, inflammation, and hyperandrogenism. Int J Mol Sci. 2022;23(8):4110. 10.3390/ijms23084110 PMC9030414 35456928

[3] Xu Y, Qiao J. Association of insulin resistance and elevated androgen levels with Polycystic Ovarian Syndrome (PCOS): a review of literature. J Healthc Eng. 2022;2022:9240569. 10.1155/2022/9240569 PMC8959968 35356614

[4] He S, Mao X, Lei H, Dong B, Guo D, Zheng B (2020). Peripheral blood inflammatory-immune cells as a predictor of infertility in women with polycystic ovary syndrome. J Inflamm Res.

[5] Rudnicka E, Suchta K, Grymowicz M, et al. Chronic low grade inflammation in pathogenesis of PCOS. Int J Mol Sci. 2021;22(7):3789. 10.3390/ijms22073789 PMC8038770 33917519

[6] Deligeoroglou E, Vrachnis N, Athanasopoulos N, Iliodromiti Z, Sifakis S, Iliodromiti S (2012). Mediators of chronic inflammation in polycystic ovarian syndrome. Gynecol Endocrinol.

[7] Brännström M, Norman RJ (1993). Involvement of leukocytes and cytokines in the ovulatory process and corpus luteum function. Hum Reprod.

[8] Chen H, Zhang Y, Li S, Tao Y, Gao R, Xu W (2021). The Association between genetically predicted systemic inflammatory regulators and polycystic ovary syndrome: a mendelian randomization study. Front Endocrinol (Lausanne).

[9] Boots CE, Jungheim ES (2015). Inflammation and human ovarian Follicular Dynamics. Semin Reprod Med.

[10] González F, Considine RV, Abdelhadi OA, Acton AJ (2019). Oxidative stress in response to Saturated Fat Ingestion is linked to insulin resistance and hyperandrogenism in polycystic ovary syndrome. J Clin Endocrinol Metab.

[11] Su NJ, Ma J, Feng DF, Zhou S, Li ZT, Zhou WP (2018). The peripheral blood transcriptome identifies dysregulation of inflammatory response genes in polycystic ovary syndrome. Gynecol Endocrinol.

[12] Shamsi M, Nejati V, Najafi G, Pour SK (2020). Protective effects of licorice extract on ovarian morphology, oocyte maturation, and embryo development in pcos-induced mice: an experimental study. Int J Reprod Biomed.

[13] Shamsi M, Nejati V, Najafi G. Therapeutic effects of licorice extract on in vitro maturation and in vitro fertilization in mice model of polycystic ovary syndrome. J Maz Univ Med Sci. 2016;25(132):113–21.

[14] Nantia EA, Moundipa PF, Monsees TK, Carreau S (2009). Medicinal plants as potential male anti-infertility agents: a review. Basic Clin Androl.

[15] Lang Q, Yidong X, Xueguang Z, Sixian W, Wenming X, Tao Z (2019). ETA-mediated anti-TNF-α therapy ameliorates the phenotype of PCOS model induced by letrozole. PLoS ONE.

[16] El Mihyaoui A, da Silva JCG, Charfi S, Candela Castillo ME, Lamarti A, Arnao MB. Chamomile (*Matricaria chamomilla* L.): a review of ethnomedicinal use, phytochemistry and pharmacological uses. Life (Basel). 2022;12(4):479. 10.3390/life12040479 PMC9032859 35454969

[17] Farideh ZZ, Bagher M, Ashraf A, Akram A, Kazem M. Effects of chamomile extract on biochemical and clinical parameters in a rat model of polycystic ovary syndrome. J Reprod Infertil. 2010;11(3):169–74. PMC3719301 23926485

[18] Heidary M, Yazdanpanahi Z, Dabbaghmanesh MH, Parsanezhad ME, Emamghoreishi M, Akbarzadeh M (2018). Effect of chamomile capsule on lipid- and hormonal-related parameters among women of reproductive age with polycystic ovary syndrome. J Res Med Sci.

[19] Alahmadi AA, Alzahrani AA, Ali SS, Alahmadi BA, Arab RA, El-Shitany NAEA (2020). Both matricaria chamomilla and metformin extract improved the function and histological structure of thyroid gland in polycystic ovary syndrome rats through antioxidant mechanism. Biomolecules.

[20] El Haouari M, Rosado JA, Phytochemical (2018). Anti-diabetic and Cardiovascular Properties of Urtica dioica L. (Urticaceae): a review. Mini-Reviews Med Chem.

[21] Bhusal KK, Magar SK, Thapa R, Lamsal A, Bhandari S, Maharjan R (2022). Nutritional and pharmacological importance of stinging nettle (Urtica dioica L.): a review. Heliyon.

[22] Farag MA, Weigend M, Luebert F, Brokamp G, Wessjohann LA (2013). Phytochemical, phylogenetic, and anti-inflammatory evaluation of 43 Urtica accessions (stinging nettle) based on UPLC-Q-TOF-MS metabolomic profiles. Phytochemistry.

[23] Taheri Y, Quispe C, Herrera-Bravo J, Sharifi-Rad J, Ezzat SM, Merghany RM (2022). *Urtica dioica*-Derived Phytochemicals for pharmacological and therapeutic applications. Evidence-Based Complement Altern Med.

[24] Bandariyan E, Mogheiseh A, Ahmadi A (2021). The effect of lutein and Urtica dioica extract on in vitro production of embryo and oxidative status in polycystic ovary syndrome in a model of mice. BMC Complement Med Ther.

[25] Najafipour F, Rahimi AO, Mobaseri M, Agamohamadzadeh N, Nikoo AS, Aliasgharzadeh A. Therapeutic effects of stinging nettle (*Urtica dioica*) in women with Hyperandrogenism. Int J Current Res Acad Rev. 2014;2(7):153–60.

[26] Hajaji S, Jabri MA, Alimi D, Rekik M, Akkari H (2019). Chamomile methanolic extract mitigates small bowel inflammation and ROS overload related to the intestinal nematodes infection in mice. Acta Parasitol.

[27] Doukkali Z, Taghzouti K, Bouidida HLH, Nadjmouddine M, Cherrah Y, Alaoui K (2015). Evaluation of anxiolytic activity of methanolic extract of Urtica urens in a mice model. Behav Brain Funct.

[28] OCED: Test No. 425: acute oral toxicity: up-and-down procedure, OECD guidelines for the testing of chemicals, section 4. In: 2008; Paris. OECD Publishing.

[29] Cora MC, Kooistra L, Travlos G (2015). Vaginal cytology of the Laboratory Rat and Mouse. Toxicol Pathol.

[30] Luo LL, Huang J, Fu YC, Xu JJ, Qian YS (2008). Effects of tea polyphenols on ovarian development in rats. J Endocrinol Invest.

[31] Chen MJ, Chou CH, Chen SU, Yang WS, Yang YS, Ho HN (2015). The effect of androgens on ovarian follicle maturation: dihydrotestosterone suppress FSH-stimulated granulosa cell proliferation by upregulating PPARÎ 3-dependent PTEN expression. Sci Rep.

[32] Wu G, Hu X, Ding J, Yang J (2020). The effect of glutamine on Dehydroepiandrosterone-induced polycystic ovary syndrome rats. J Ovarian Res.

[33] Dou L, Zheng Y, Li L, Gui X, Chen Y, Yu M (2018). The effect of cinnamon on polycystic ovary syndrome in a mouse model. Reprod Biol Endocrinol.

[34] Fukuda S, Orisaka M, Tajima K, Hattori K, Kotsuji F (2009). Luteinizing hormone-induced akt phosphorylation and androgen production are modulated by MAP kinase in bovine theca cells. J Ovarian Res.

[35] Li T, Mo H, Chen W, Li L, Xiao Y, Zhang J et al. Role of the PI3K-Akt signaling pathway in the pathogenesis of polycystic ovary syndrome. Reprod Sci. 2017;24(5):646–55. 10.1177/1933719116667606 27613818

[36] Restuccia DF, Hynx D, Hemmings BA (2012). Loss of PKBβ/Akt2 predisposes mice to ovarian cyst formation and increases the severity of polycystic ovary formation in vivo. DMM Dis Model Mech.

[37] Shah KN, Patel SS (2016). Phosphatidylinositide 3-kinase inhibition: a new potential target for the treatment of polycystic ovarian syndrome. Pharm Biol.

[38] Zuo T, Zhu M, Xu W. Roles of oxidative stress in polycystic ovary syndrome and cancers.Oxid Med Cell Longev. 2016;2016:8589318. 10.1155/2016/8589318 PMC4684888 26770659

[39] Agarwal A, Gupta S, Sharma RK. Role of oxidative stress in female reproduction. Reprod Biol Endocrinol. 2005;3:28. 10.1186/1477-7827-3-28 PMC1215514 16018814

[40] Paixão L, Ramos RB, Lavarda A, Morsh DM, Spritzer PM. Animal models of hyperandrogenism and ovarian morphology changes as features of polycystic ovary syndrome: a systematic review. Reprod Biol Endocrinol. 2017;15(1):12. 10.1186/s12958-017-0231-z PMC5301391 28183310

[41] Grzesiak M, Kapusta K, Kaminska K, Palka S, Kmiecik M, Zubel-Lojek J (2021). Effect of dietary supplementation with nettle or fenugreek on folliculogenesis and steroidogenesis in the rabbit ovary – an in vivo study. Theriogenology.

[42] Murasawa M, Takahashi T, Nishimoto H, Yamamoto S, Hamano S, Tetsuka M (2005). Relationship between ovarian weight and follicular population in heifers. J Reprod Dev.

[43] Alahmadi AA, Alahmadi BA, Wahman LF, El-Shitany NA (2021). Chamomile flower extract ameliorates biochemical and histological kidney dysfunction associated with polycystic ovary syndrome. Saudi J Biol Sci.

[44] Zhang R, Liu H, Bai H, Zhang Y, Liu Q, Guan L (2017). Oxidative stress status in chinese women with different clinical phenotypes of polycystic ovary syndrome. Clin Endocrinol (Oxf).

[45] Bi X, Soong YY, Lim SW, Henry CJ (2015). Evaluation of antioxidant capacity of chinese five-spice ingredients. Int J Food Sci Nutr.

[46] Hajizadeh-Sharafabad F, Varshosaz P, Jafari-Vayghan H, Alizadeh M, Maleki V. Chamomile (Matricaria recutita L.) and diabetes mellitus, current knowledge and the way forward: A systematic review. Complement Ther Med. 2020. 10.1016/j.ctim.2019.102284 31987240

[47] Pourahmadi M, Jashni HK, Maryam B, Jahromi AS. The effect of hydro-alcoholic extract of urtica dioica root on testes in adult rats. Life Sci J. 2014.

[48] Koch E. Extracts from fruits of saw palmetto (Sabal serrulata) and roots of stinging nettle (Urtica dioica): Viable alternatives in the medical treatment of benign prostatic hyperplasia and associated lower urinary tracts symptoms. Planta Medica. 2001. 10.1055/s-2001-16496 11509966

[49] Golalipour MJ, Ghafari S, Afshar M (2010). Protective role of Urtica dioica L. (Urticaceae) extract on hepatocytes morphometric changes in STZ diabetic Wistar rats. Turkish J Gastroenterol.

[50] Luther JM, Brown NJ. The renin-angiotensin-aldosterone system and glucose homeostasis. Trends Pharmacol Sci. 2011;32(12):734–9. 10.1016/j.tips.2011.07.006 PMC3223326 21880378

[51] Gourdy P, Bourgeois EA, Levescot A, Pham L, Riant E, Ahui ML (2016). Estrogen therapy delays autoimmune diabetes and promotes the protective efficiency of natural killer T-cell activation in female nonobese diabetic mice. Endocrinology.

[52] Pennell LM, Galligan CL, Fish EN. Sex affects immunity. J Autoimmun. 2012;38(2–3):J282–91. 10.1016/j.jaut.2011.11.013 22225601

[53] Walecki M, Eisel F, Klug J, Baal N, Paradowska-Dogan A, Wahle E (2015). Androgen receptor modulates Foxp3 expression in CD4 + CD25 + Foxp3 + regulatory T-cells. Mol Biol Cell.

[54] Nasri F, Doroudchi M, Jahromi BN, Gharesi-Fard B. T helper cells profile and cd4 + cd25 + foxp3 + regulatory t cells in polycystic ovary syndrome. Iran J Immunol. 2018;15(3):175–85. 10.22034/IJI.2018.39387 30246693

[55] Krishna MB, Joseph A, Subramaniam AG, Gupta A, Pillai SM, Laloraya M (2015). Reduced tregs in peripheral blood of PCOS patients - a consequence of aberrant Il2 signaling. J Clin Endocrinol Metab.

[56] Zhao Y, Zhang C, Huang Y, Yu Y, Li R, Li M (2015). Up regulated expression of wnt5a increases inflammation and oxidative stress via PI3K/AKT/NFêB signaling in the granulosa cells of PCOS patients. J Clin Endocrinol Metab.

[57] Benson S, Janssen OE, Hahn S, Tan S, Dietz T, Mann K (2008). Obesity, depression, and chronic low-grade inflammation in women with polycystic ovary syndrome. Brain Behav Immun.

[58] Hatzirodos N, Bayne RA, Irving-Rodgers HF, Hummitzsch K, Sabatier L, Lee S (2011). Linkage of regulators of TGF‐β activity in the fetal ovary to polycystic ovary syndrome. FASEB J.

[59] Amato G, Conte M, Mazziotti G, Lalli E, Vitolo G, Tucker AT (2003). Serum and follicular fluid cytokines in polycystic ovary syndrome during stimulated cycles. Obstet Gynecol.

[60] Nissi R, Talvensaari-Mattila A, Kotila V, Niinimäki M, Järvelä I, Turpeenniemi-Hujanen T (2013). Circulating matrix metalloproteinase MMP-9 and MMP-2/TIMP-2 complex are associated with spontaneous early pregnancy failure. Reprod Biol Endocrinol.

[61] Dambala K, Vavilis D, Bili E, Goulis DG, Tarlatzis BC (2017). Serum visfatin, vascular endothelial growth factor and matrix metalloproteinase-9 in women with polycystic ovary syndrome. Gynecol Endocrinol.

[62] Raja-Khan N, Urbanek M, Rodgers RJ, Legro RS. The role of TGF-β in polycystic ovary syndrome. Reprod Sci. 2014;21(1):20–31. 10.1177/1933719113485294 PMC5933191 23585338

[63] Sproul K, Jones MR, Mathur R, Azziz R, Goodarzi MO (2010). Association study of four key folliculogenesis genes in polycystic ovary syndrome. BJOG An Int J Obstet Gynaecol.

